# Sumoylation of the Carboxy-Terminal of Human Cytomegalovirus DNA Polymerase Processivity Factor UL44 Attenuates Viral DNA Replication

**DOI:** 10.3389/fmicb.2021.652719

**Published:** 2021-04-21

**Authors:** Jun Chen, Guanlie Li, Haiqing He, Xin Li, Wenjing Niu, Di Cao, Ao Shen

**Affiliations:** ^1^Key Laboratory of Molecular Target and Clinical Pharmacology, State Key Laboratory of Respiratory Disease, School of Pharmaceutical Sciences and The Fifth Affiliated Hospital, Guangzhou Medical University, Guangzhou, China; ^2^Department of Biotechnology, College of Life Science and Technology, Jinan University, Guangzhou, China

**Keywords:** human cytomegalovirus, UL44, sumoylation, DNA replication, UBC9

## Abstract

Controlled regulation of genomic DNA synthesis is a universally conserved process for all herpesviruses, including human cytomegalovirus (HCMV), and plays a key role in viral pathogenesis, such as persistent infections. HCMV DNA polymerase processivity factor UL44 plays an essential role in viral DNA replication. To better understand the biology of UL44, we performed a yeast two-hybrid screen for host proteins that could interact with UL44. The most frequently isolated result was the SUMO-conjugating enzyme UBC9, a protein involved in the sumoylation pathway. The UBC9-UL44 interaction was confirmed by *in vitro* His-tag pull-down and *in vivo* co-immunoprecipitation assays. Using deletion mutants of UL44, we mapped two small regions of UL44, aa 11–16, and 260–269, which might be critical for the interaction with UBC9. We then demonstrated that UL44 was a target for sumoylation by *in vitro* and *in vivo* sumoylation assays, as well as in HCMV-infected cells. We further confirmed that ^410^lysine located within a ψKxE consensus motif on UL44 carboxy-terminal was the major sumoylation site of UL44. Interestingly, although ^410^lysine had no effects on subcellular localization or protein stability of UL44, the removal of ^410^lysine sumoylation site enhanced both viral DNA synthesis in transfection-replication assays and viral progeny production in infected cells for HCMV, suggesting sumoylation can attenuate HCMV replication through targeting UL44. Our results suggest that sumoylation plays a key role in regulating UL44 functions and viral replication, and reveal the crucial role of the carboxy-terminal of UL44, for which little function has been known before.

## Introduction

Human cytomegalovirus (HCMV), a member of β-herpesviruses, is the leading viral cause of congenital abnormalities and intellectual disability in newborns, and it also causes severe life-threatening complications in immunocompromised individuals like AIDS patients and transplant recipients (Mocarski et al., 2007). The process of genomic DNA replication is highly conserved across all herpesviruses and is the target for most of the current FDA-approved anti-herpes therapeutic agents (Biron, 2006; Piret and Boivin, 2014). Understanding the mechanism of herpesviral genomic DNA replication is important for the development of new strategies to treat infections of these viruses including HCMV. The replication of viral double-stranded DNA genome occurs in the nuclei of infected cells via a rolling circle process, mediated by the activities of a conserved set of six viral proteins which includes the DNA polymerase UL54, the polymerase processivity factor UL44, the single-stranded DNA-binding protein UL57, the helicase UL105, the primase UL70, and the primase-associated factor UL102 (Ertl and Powell, 1992; Mocarski et al., 2007; Weiland et al., 1994).

UL44 forms dimer, binds double-stranded DNA and UL54 specifically, and stimulates long chain DNA synthesis efficiently (Appleton et al., 2004; Ertl and Powell, 1992; Weiland et al., 1994). Full length UL44 contains 433 amino acids (aa), and the crystal structure of its amino-terminal segment (aa 1–290) was solved (Appleton et al., 2004). This structure consists of two topologically similar subdomains connected by a loop running lengthways, and shows a remarkable similarity to that of other DNA polymerase processivity factors such as herpes simplex virus (HSV) UL42 and the sliding clamp PCNA (Krishna et al., 1994; Zuccola et al., 2000; Appleton et al., 2004). Interestingly, the amino-terminal segment of UL44 is also responsible for all activities of UL44 as a processivity factor, as deletion of aa 291–433 does not alter any of its known biochemical activities *in vitro* (Weiland et al., 1994; Appleton et al., 2004; Appleton et al., 2006). In contrast, little is known about the role of the carboxy-terminal segment of UL44 other than it contains a nuclear localization signal (NLS) (Alvisi et al., 2005; Silva et al., 2010). Notably, UL44 carboxy-terminal was found to be indispensable for virus replication and for the formation of DNA replication compartments in infected cells (Kim and Ahn, 2010; Silva et al., 2010), even when this segment was replaced with another NLS that ensured nuclear localization (Silva et al., 2010). This clearly argues for a crucial role for the carboxy-terminal of UL44 in HCMV viral DNA synthesis and viral replication, beyond its role in nuclear localization. It was proposed that the carboxy-terminal segment might interact with host or viral proteins involved in DNA replication (Kim and Ahn, 2010), however, the exact role of the carboxy-terminal of UL44 remains to be elucidated thus far.

Great efforts have been made to assess features of human-HCMV interactions, and host responses to HCMV infection. During infection, HCMV manipulates hosts in various ways to mediate its own replication, transcription or immune evasion. Post-translational modifications are unique mechanisms that allow cells and virus to specifically modify activities or interactions of key proteins. For example, most tegument proteins of HCMV are phosphorylated and are highly immunogenic (Mocarski et al., 2007; Chang et al., 2016). Covalent linkage of small ubiquitin-related modifier (SUMO) to its substrate, termed as sumoylation, is an important post-translational modification mechanism by which the functions of many cellular proteins are regulated. Sumoylation has roles in the control of protein stability, activity and subcellular localization, and is involved in transcriptional regulation, gene expression, chromatin structure, nuclear transport and RNA metabolism (Hay, 2005; Bossis and Melchior, 2006). While sumoylation is known to play a key role in the regulation of host defense to virus infection, herpesviruses also usurp sumoylation against the host’s intrinsic and innate immune response to favor their pathogenesis (Boggio and Chiocca, 2006; Chang et al., 2016; Gan et al., 2016).

At least three SUMO proteins have been described in mammals, among them, conjugation of SUMO-1 has been shown to play a functional role in a number of biological processes, whereas the role of SUMO-2 and SUMO-3 modification is less clear (Hay, 2005; Bossis and Melchior, 2006). Sumoylation is controlled by an enzymatic cascade in an analogous fashion to ubiquitination: SUMO is first activated by the E1 SUMO-activating enzyme UBA2/AOS1, then transferred to the E2 SUMO-conjugating enzyme UBC9, and finally covalently attached to lysine residues of a target protein with or without the aid of E3 ligases (Hay, 2005; Bossis and Melchior, 2006). Typically, lysine residues subject to SUMO modification are found within a consensus sumoylation motif ψKxE (usually ψ is a hydrophobic residue, x is any residue and K is the conjugation site) (Rodriguez et al., 2001; Sampson et al., 2001), but modifications at non-consensus sites have also been reported (Comerford et al., 2003; Yan et al., 2007).

Sumoylation is linked, both positively and negatively, with the replication of many different viruses both in terms of modification of viral proteins and modulation of sumoylated cellular proteins that influence the efficiency of infection (Boggio and Chiocca, 2006; Chang et al., 2016; Gan et al., 2016; Yang et al., 2017). Interestingly, so far most viral proteins being reported to interplay with the sumoylation pathway are encoded by herpesviruses and belong to immediate-early proteins, which include but not limited to: HCMV IE1 and IE2, HSV-1 ICP0, Epstein-Barr virus Zta and Rta, human herpes virus-6 IE1, and Kaposi’s sarcoma-associated herpesvirus K-bZIP and LANA (Muller and Dejean, 1999; Hofmann et al., 2000; Ahn et al., 2001; Gravel et al., 2002; Chang et al., 2004; Nevels et al., 2004; Adamson, 2005; Izumiya et al., 2005; Boggio and Chiocca, 2006; Gan et al., 2016; Lin et al., 2017; Reuter et al., 2017; Jan Fada et al., 2020). Though consequences of sumoylation on those viral proteins are different and not yet fully understood, it is believed that sumoylation is one of the ways for herpesviruses to regulate their immediate-early transcriptional activators and/or against host intrinsic immune response. However, the effects of sumoylation on other herpesviral proteins, especially its influence during the early and late phase of herpesviral infection, remain largely unknown.

In an effort to gain a better understanding of the HCMV DNA replication process, we carried out a series of yeast two-hybridscreens to identify host proteins that interact with HCMV replication core proteins. We reported that several cellular factors participate in HCMV DNA replication process via modulating subcellular distribution of viral proteins in infected cells (Shen et al., 2011; Pei et al., 2012; Luo et al., 2013). In this study we further identified the SUMO-conjugating enzyme UBC9 as a UL44 binding partner. The interaction was confirmed by *in vitro* His-tag pull-down and *in vivo* co-immunoprecipitation (co-IP) assays. Two small regions located at aa 11–16 and 260–269, corresponding to the first and last β-strands of UL44 core, respectively, were likely critical for the interaction of UL44 with UBC9. We then demonstrated that UL44 was a target for sumoylation by *in vitro* and *in vivo* sumoylation assays, as well as in HCMV-infected cells. A previous study reported UL44 could be extensively sumoylated at multiple sites, but the effect of sumoylation on UL44 had not been examined in the study due to expression issue after mutagenesis (Sinigalia et al., 2012). In contrast to their report, we found that ^410^lysine, located at the extreme carboxy terminus of UL44 and within a ψKxE consensus sumoylation motif, was the major sumoylation site of the protein. More importantly, although the mutation of ^410^lysine had no effects on subcellular localization or protein stability of UL44, we found that the removal of ^410^lysine sumoylation site enhanced both viral DNA synthesis in transfection-replication assays and viral progeny production in infected cells for HCMV, indicating a negative modulation of sumoylation on HCMV replication through UL44 carboxy-terminal.

Here we identified UL44 as a conserved sumoylated herpesviral protein not belonging to immediate-early proteins, implying sumoylation is involved throughout the process of herpesviral infection. Our findings suggest that sumoylation plays an important role in HCMV DNA and virus replication, and reveal the crucial role of UL44 carboxy-terminal in regulating UL44 functions in a self-restricted way. These results provide insight into how herpesviruses utilize cellular pathways to achieve optimal persistent infections within the host through self-restricted replication.

## Materials and Methods

### Cells and Viruses

Human foreskin fibroblasts (HFFs), HeLa cells, 293T cells, and Vero cells were maintained in Dulbecco’s modified Eagle medium (DMEM) supplemented with 10% fetal bovine serum. HCMV (wild-type, mutant and revertant Towne-BAC) were generated and propagated in HFFs using a method described before (Dunn et al., 2003).

### Oligonucleotides

Oligonucleotide primers used in this study for sequencing, PCR and mutagenesis are listed in Table 1.

**TABLE 1:** Primers used in the study.

Primer	Sequence (5′–3′)	Restriction sites
GAL4AD	AATACCACTACA ATGGATG	\
T7	TAATACGACTCACTATAGGGC	\
UL44F	GATATCCATATGTTGTCGA CCGATCGCAAGACGCGCCTCTC	*Nde*I *Sal*I
UL44R	CGCGGATCCGGTACCCTAGCC GCACTTTTGCTTCTTG	*Bam*HI *Kpn*I
K410AF	ATTACTTCAACGACGCGGCA GAGGAGAGCGACAGCG	\
K410AR	CGCTGTCGCTCTCCTCTGC CGCGTCGTTGAAGTAAT	\
C93SF	CCCTTCGGGGACAGT GTCCCTGTCCAT	\
C93SR	ATGGACAGGGACACT GTCCCCGAAGGG	\
44C1F	ATCTCGAGCCGATCGCA AGACGCGCCTCT	*Xho*I
44C1R	GCGGATCCCTAGCCGCAC TTTTGCTTCTTG	*Bam*HI
UL44F1	TTACATATGTTGTCGACG GCCGTGCACGTGGATCT	*Nde*I *Sal*I
UL44F2	ATACATATGGTGTCGACT GCGCTCAAGGAGAACACC	*Nde*I *Sal*I
UL44F5	AATCATATGCTGTCGACG CCACCGACGCTGGCGCT	*Nde*I *Sal*I
UL44F6	CCTCATATGGCGTCGACG CTGAAGCCGTACAAGAC	*Nde*I *Sal*I
UL44R1	TATGGATCCGGTACCTTCC TCGGTGAGAAAGTTCT	*Bam*HI *Kpn*I
UL44R2	TATGGATCCGGTACCTTCCA GCTCGCTGCCGTTGG	*Bam*HI *Kpn*I
UL44R3	TATGGATCCGGTACCACCG TTGCGGCTGGCCAC	*Bam*HI *Kpn*I
UL44R4	TATGGATCCGGTACCC GGTAGTTTGGTGACGGC	*Bam*HI *Kpn*I
KNLSF	ATCTCGAGATTACTTCAA CGACGCGAAAG	*Xho*I
ANLSF	ATCTCGAGATTACT TCAACGACGCGGCAG	*Xho*I
NLSR	TGATCAGTTATCTAGA TCCGGTGGATCC	*Bam*HI
C1F	ATCTCGAGTCATGTCGGG GATCGCCCTC	*Xho*I
C1R	CAGGATCCTTATGAGGGCG CAAACTTCT	*Bam*HI
SUMO1F	ATGTCGACCTCTGACC AGGAGGCAAAACCT	*Sal*I
SUMO1R	ATTGCGGCCGCTTAACCCCC CGTTTGTTCCTGAT	*Not*I
UL36-38F	GCGAATTCCACTTCTTTC TTTAATTATGTC	*Eco*RI
UL36-38R	TTACTCGAGCTCGTCA TCCTCTCCTCAATC	*Xho*I
UL54F	GATATCCATATGAGAATTCTGT TTTTCAACCCGTATCTGAGC	*Eco*RI
UL54R	GATATCCATATGGAATTCTCA ACAGCATTCGTGCGCCTTG	*Eco*RI
UL57F	GATATCCATATGGCGGCCGCTA GCCACGAGGAACTAACCG	*Not*I
UL57R	CGCGGATCCGCGGCCGCTTAC AACCGGCTGCGTTTGGC	*Not*I
UL69F	CCGGAATTCTCTCGAGGTGAGC TGCACTCACGCGGCCGT	*Xho*I
UL69R	CGCGGATCCGCGGCCGCTTAGTCA TCCATATCATCGCTGTAAC	*Not*I
UL70F	GCATCATATGGGGTCGACGATGA CGCTCGTTCTGTT	*Sal*I
UL70R	GGGGATCCGGTACCGTGGAAAG TGAGGCTCAGAC	*Kpn*I
UL84F	GATATCCATATGAAGATCTCT CCACGCGCCGACCCCAACC	*Bgl*II
UL84R	CCGGAATTCGGTACCTTAGAG ATCGCCGCAGACCATG	*Kpn*I
UL102F	CCGGAATTCGCGGCCGCAA CCGCTCAGCCGCCGCTGC	*Not*I
UL102R	CGCGGATCCGCGGCCGCTTA AGCGTTGAGCCGGAAAAACC	*Not*I
UL105F	GATATCCATATGAAGATCTC TTCGATGACGGCCTCGTCATC	*Not*I
UL105R	CCGGAATTCAGATCTTCAAA AAATAAGCGTGGTGCGTT	*Bgl*II
UL112F1	TAGAGATCTTGGATCTGC CTACTACCGTCGT	*Bgl*II
UL112R1	GAGGAGAGCCGTCGTTCT CGGAGGAGGGAG	\
UL112F2	CGAGAACGACGGCTCTC CTCCCCTCCGG	\
UL112R2	ATTGGTACCTTAATCGT CGAAAAACGCCGC	*Kpn*I
IE1F	CCGAATTCAGGTCGACAATGG AGTCCTCTGCCAAGAG	*Sal*I
IE1R	TTGGATCCTCGAGTAGTTTAC TGGTCAGCCTTGC	*Xho*I
IE2F	CCGAATTCAGGTCGACAATGG AGTCCTCTGCCAAGAG	*Sal*I
IE2R	TTGGATCCAGATCTTTAC TGAGACTTGTTCCTCAGG	*Bgl*II
SV40F	GCAGATCTTTTAGGTGAC ACTATAG	*Bgl*II
SV40R	GCAAGCTTCCTCCAA AAAAGCCTCT	*Hin*dIII

### Construction of Plasmids

Full length HCMV UL44 gene was amplified by PCR using the primers UL44F/UL44R and a bacterial artificial chromosome-based clone of HCMV Towne strain (Towne-BAC) (Dunn et al., 2003) as template, digested with the respective restriction enzyme incorporated into the primers, then inserted into *Nde*I/*Bam*HI digested pGBKT7 (Clontech) containing a fusion GAL4 DNA-binding domain as bait for library screen (designated as pGBKT7-UL44), and *Nde*I/*Bam*HI digested pET-28a (Novagen) with a N-terminal His tag for prokaryotic expression (designated as pET-28a-UL44), and *Sal*I/*Kpn*I digested pCMV-HA (Clontech) with a N-terminal hemagglutinin (HA) tag for mammalian expression (designated as pCMV-HA-UL44). Truncated UL44 mutants UL44Δ1, Δ6, Δ7 and Δ8 were constructed by digestion of pGBKT7-UL44 plasmid with *Nde*I/*Nco*I, *Nde*I/*Eco*RI, *Nco*I/*Bam*HI, and *Eco*RI/*Bam*HI, and then inserted into pGBKT7 vector with same digestion, respectively (designated as pGBKT7-UL44Δ1, Δ6, Δ7, and Δ8). Truncated UL44 mutants UL44Δ2, Δ3, Δ4, Δ5, Δ9, Δ10, Δ11 and Δ12 were made by PCR using pGBKT7-UL44 as a template with respective primers T7/UL44R1, T7/UL44R3, T7/UL44R4, T7/UL44R2, UL44F1/UL44R1, UL44F2/UL44R1, UL44F6/UL44R1, and UL44F5/UL44R1, then inserted into *Nde*I/*Bam*HI digested pGBKT7 (designated as pGBKT7-UL44Δ2, Δ3, Δ4, Δ5, Δ9, Δ10, Δ11, and Δ12). Their corresponding subclones to pCMV-HA were made by insertion into *Sal*I/*Kpn*I digested pCMV-HA. pCMV-HA-UL44-K410A mutant, in which the ^410^lysine residue was mutated to ^410^alanine, was generated by Quikchange Site-direct Mutagenesis Kit (Stratagene) using the complementary primers K410AF/K410AR and pCMV-HA-UL44 as template.

Wild-type or K410A mutated UL44 gene was amplified by PCR using the primers 44C1F/44C1R, and pCMV-HA-UL44 or pCMV-HA-UL44-K410A as template, then inserted into *Xho*I/*Bam*HI digested pEGFP-C1 (Clontech) with a N-terminal fusion EGFP (designated as pEGFP-UL44WT and pEGFP-UL44-K410A). Plasmids expressing the last 29 amino acids of UL44 fused to EGFP, which contain a ψKxE consensus sumoylation motif and UL44 nuclear localization signal (NLS) motif, was generated by PCR using the primers KNLSF/NLSR, ANLSF/NLSR, and pEGFP-UL44WT or pEGFP-UL44-K410A as template, then inserted into *Xho*I/*Bam*HI digested pEGFP-C1 (designated as pEGFP-NLS_*UL*44*WT*_ and pEGFP-NLS_*UL*44–*K*410*A*_). The nanobody expressing construct pCMV-Myc-NB80 was kindly provided by Dr. Yang K. Xiang (UC-Davis).

pACT2-UBC9 containing full length UBC9 gene was extracted from human fetal brain cDNA library (Clontech), and the insert was subcloned into *Eco*RI/*Xho*I digested pRK-7-FLAG (kindly provided by Dr. Hongbin Shu, Wuhan University) or pCMV-Myc (Clontech) with a N-terminal FLAG or Myc tag (designated as pRK-7-UBC9 and pCMV-Myc-UBC9). pDsRed-UBC9 with a N-terminal red fluorescent fusion protein was amplified by PCR from pACT2-UBC9 using the primers C1-F/C1-R and inserted into *Xho*I/*Bam*HI digested pDsRed-monomer-C1 (Clontech). pCMV-Myc-UBC9-DN mutant, in which the ^93^cysteine residue was mutated to ^93^serine, was generated by Quikchange Site-direct Mutagenesis Kit (Stratagene) using the complementary primers C93SF/C93SR and pCMV-Myc-UBC9 as template, and further subcloned to pRK-7-FALG (designated as pRK-7-UBC9-DN). pCMV-Myc-SUMO-1 that encode active form of SUMO-1 with Myc tag were generated by PCR using human cDNA library as template and the primers SUMO1F/-R. Full length HCMV genes UL54, UL57, UL69, UL70, UL84, UL102, and UL105 were amplified by PCR using the primers listed in Table 1 and the DNA of Towne-BAC as template, digested with the respective restriction enzyme incorporated into the primers, then inserted into digested pCMV-HA or pCMV-Myc vector. A plasmid that expressed gene products of HCMV UL36-38 locus was generated by PCR using the same method described above, and inserted into pcDNA3.1(+) vector (Invitrogen). The plasmids that contained the sequences encoding HCMV IE1-72 or IE2-86 were generated by PCR from pCGN-IE1 or pCGN-IE2 plasmid (kindly provided by Dr. Thomas Shenk, Princeton University) and digested with the restriction enzyme incorporated into the primers, then inserted into digested pCMV-HA vector. The HCMV IRS1 expression construct pEQ890 (kindly provided by Dr. Adam Geballe, University of Washington) and was described previously (Hakki et al., 2006). To generate a plasmid that expressed HCMV UL112-113 gene product p84, pGBKT7-UL112ex1 and pGBKT7-UL112ex2 which have been previously described (To et al., 2011) were used as PCR templates, and UL112 exon1 and exon2 coding sequences were separately amplified using the primers UL112F1/UL112R1 and UL112F2/UL112R2, respectively, PCR products were then mixed and followed by standard overlapping PCR method. The replication reporter plasmid pGL3-14 which contained the *Pvu*II/*Kpn*I segment (5,073 bp) of HCMV Towne strain harboring viral oriLyt was made as detailed below: First, a sequence spanning the SV40 early promoter was amplified by PCR from pRK-7-FLAG vector using the primers SV40F/SV40R, the PCR product was digested with *Bgl*II/*Hin*dIII and then inserted into pGL3-basic vector (Promega) thus generating the resultant vector pGL3-SV40. Second, the DNA of Towne-BAC was digested with *Hin*dIII and resolved on 0.8% agarose gel, then the HCMV *Hin*dIII-D fragment was extracted using E.Z.N.A. Gel Extraction Kit (Omega Bio-Tek) and further digested with *Pvu*II/*Kpn*I. Finally, the *Pvu*II/*Kpn*I HCMV oriLyt segment was resolved on 0.8% agarose gel and inserted into the *Kpn*I/*Sma*I site of pGL3-SV40, generating resultant plasmid pGL3-14. The plasmids used for transfection-replication assay are listed in Table 2. All constructs and integrity of plasmids used in this study were confirmed by both restriction digestion profile and DNA sequencing.

**TABLE 2:** HCMV DNA replication-related genes and plasmids used in the study.

Plasmids	HCMV genes	Functions
pCMV-HA-UL44	UL44	Polymerase processivity factor
pCMV-Myc-UL54	UL54	DNA polymerase
pCMV-Myc-UL57	UL57	Single-stranded DNA-binding
pCMV-Myc-UL70	UL70	Primase
pCMV-Myc-UL102	UL102	Primase-associated factor
pCMV-Myc-UL105	UL105	Helicase
pcDNA3.1-UL3638	UL36-38	Regulatory, inhibition of apoptosis
pCMV-Myc-UL69	UL69	Posttranscriptional regulator, cell cycle modulation
pCMV-HA-UL84	UL84	Initiation of oriLyt-specific DNA replication
pCMV-Myc-UL112	UL112	Transcriptional activator
pEQ890	IRS1	Transcriptional activator
pCMV-HA-IE1	IE1-72	Enhances activation by IE2
pCMV-HA-IE2	IE2-86	Regulation of viral and host gene expression, cell cycle modulation
pGL3-14	oriLyt	The single origin of DNA replication

### Yeast Two-Hybrid Screening and Assays

*Saccharomyces cerevisiae* strain AH109, human fetal brain cDNA library that was constructed on pACT2 fused with the GAL4 activation domain, and control vectors pGADT7, pGADT7-T, pGBKT7-p53, and pGBKT7-Lam were from Clontech. pGBKT7-UL44 was first introduced into strain AH109 and verified that do not activate the report genes. Then yeast strain containing pGBKT7-UL44 was transformed with the library. Positive clones were selected on synthetic dropout medium lacking four nutrients tryptophan, leucine, adenine, and histidine (QDO) and tested for β-galactosidase activity by colony-lift filter assay according to the manufacturer’s instructions. Library plasmids from positive colonies were rescued by transformation of *E. coli* DH5α with plasmids extracted from yeast using E.Z.N.A. Yeast Plasmid Kit (Omega Bio-Tek), cotransformated into yeast AH109 with pGBKT7-UL44 or empty bait vector pGBKT7 as control, plated on synthetic dropout medium lacking tryptophan and leucine (DDO), then restreaked colonies on QDO and subjected to β-galactosidase activity test. The insert of library plasmid were subsequently sequenced by GAL4AD primer and were characterized by a search for homologies in the National Center for Biotechnology Information databases. To map the UBC9-interacting domain of UL44, deletion derivatives of UL44 (pGBKT7-UL44Δ1 to Δ12) were cotransformed with pACT2-UBC9 into AH109 cells, plated on DDO then restreaked colonies on QDO and subjected to β-galactosidase activity test also.

### Fluorescent Localization

pDsRed-UBC9 and pEGFP-UL44 were transiently cotransfected into Hela cells using Lipofectamine 2000 (Invitrogen). After 48 h, cells were first stained with Hoechst 33258 dye (Sigma-Aldrich) to visualize nuclei, then fluorescent signals were analyzed with the Olympus FluoView FV1000 laser confocal microscope. To investigate the effect of sumoylation on UL44 subcellular location, pEGFP-UL44WT, pEGFP-UL44-K410A, pEGFP-NLS_*UL*44*WT*_, pEGFP-NLS_*UL*44–*K*410*A*_, and pRK-7-UBC9-DN were transfected into Hela cells alone or together as indicated, same treated as described above and then analyzed with the Leica TCS_SP2 laser confocal microscope.

### His-Tag Pull-Down Assay

pET-28a-UL44 was transformed into *E. coli* BL21(DE3) and induced by IPTG to express His-UL44 fusion protein. pCMV-Myc-UBC9 was transfected into 293T cells, the mammalian cell lysates were prepared after 48 h and used as the source of Myc-tagged UBC9 protein. His-tag pull-down experiments were performed using Pierce His Protein Interaction Pull-Down Kit following the manufacturer’s protocol (Thermo Fisher Scientific) supplemented with protease inhibitor cocktail (Roche), and 10 mM β-mercaptoethanol was added into both lysis and elution buffer to prevent the aggregation of His-UL44. The bound proteins were analyzed by western blot using anti-His and anti-Myc antibodies (Sigma-Aldrich).

### Co-immunoprecipitation

To test the interaction of UL44 with UBC9, 293T cells were transiently transfected with pCMV-HA-UL44 (full length or deletion mutants) and pCMV-Myc-UBC9, or pCMV-Myc-NB80 as negative control. After 48 h cells were harvested and co-IP experiments were performed using either Pierce c-Myc-Tag IP/Co-IP Kit or Pierce HA Tag IP/Co-IP Kit following the manufacturer’s protocol (Thermo Fisher Scientific) supplemented with protease inhibitor cocktail (Roche). The bound proteins were analyzed by western blot using anti-HA and anti-Myc antibodies (Sigma-Aldrich).

### *In vitro* Sumoylation Analysis

Bacterially expressed His-tagged UL44, and SUMO-activating E1 enzyme, UBC9, UBC9 dominant negative, SUMO-1 (Boston Biochem) were used to examine UL44 sumoylation *in vitro*. Each SUMO conjugation reaction (20 μl) contained 1 μg of purified His-UL44, 300 ng each of UBC9 (or UBC9 dominant negative), 500 ng SUMO-1, 15 ng of E1 enzyme, and 5ul of a buffer containing 200 mM Tris-HCl, pH 7.8, 20 mM MgCl2, 20 mM ATP, 4 mM DTT, and 800 μM N-ethylmaleimide. Reaction mixtures were incubated at 37°C for 2 h. After incubation, 5 μl of sample buffer was added to the reaction mixtures, which were then heated at 95°C for 5 min. Proteins were immediately separated by SDS-PAGE electrophoresis. UL44 was then detected by western blot with anti-His antibody.

### *In vivo* Sumoylation Analysis

293T cells were transiently transfected with pCMV-HA-UL44 (wild-type or K410A mutant), pCMV-Myc-SUMO-1, pRK-7-UBC9 or pRK-7-UBC9-DN as indicated. Forty eight hours later, cells were resuspended in SDS sample buffer and boiled for 5 min, after reducing the viscosity of samples by sonication and centrifuged for 10 min at 14,000 g, the supernatants were analyzed by western blot using anti-HA antibody. The supernatants were 50-folds diluted before they were analyzed by anti-Myc antibody, in order to reduce the background caused by cellular proteins which were also modified by exogenous Myc-SUMO.

To test the conjugation of UL44 with SUMO-1, 293T cells were transiently transfected with pCMV-HA-UL44 and pCMV-Myc-SUMO-1 alone or together. After 48 h cells were lysed in SDS sample buffer (5% SDS, 0.15 M Tris-HCl [pH 6.7], 30% glycerol) and then diluted 1:10 in PBS/0.5% NP40 plus protease inhibitor cocktail and 10 μM iodoacetamide (IAM) before incubation for 3 h at 4°C with the anti-HA antibody coated beads. The beads were collected, washed 3 times with ice-cold PBS/0.5% NP40 plus protease inhibitor cocktail and 10 μM IAM, and the complexes were recovered by boiling in electrophoresis sample buffer. Both lysates and IPs were probed with anti-HA and anti-Myc antibodies.

### Pulse-Chase Protein Stability Test

HFFs were infected with wild-type HCMV (UL44WT-Zeo) or HCMV carrying UL44-K410A mutant (UL44-K410A-Zeo) at an MOI of 1. At 48 h post-infection, cells were washed twice with pre-warmed PBS before being starved 1 h in methionine-free DMEM (Life Technologies). Cells were then pulsed for 4 h in methionine-free DMEM supplemented with 50 μM methionine analog Click-iT AHA (Thermo Fisher Scientific). After pulse labeling, cells were either immediately harvested (0 h time point), or washed twice in pre-warmed PBS before being chased in regular DMEM supplemented with 2 mM L-methionine (Sigma-Aldrich) for 6 h before harvest (6 h time point). Cell lysates containing AHA-incorporated proteins were preclear with Protein-A beads and then immunoprecipitated with anti-UL44 antibody (Santa Cruz Biotechnology) or mouse IgG as IP control, same amount of lysate from each time point determined by BCA assay was used. The IPs were further cross-linked with Biotin Alkyne (Thermo Fisher Scientific) using Click-iT Protein Reaction Buffer Kit (Thermo Fisher Scientific) according to manufacturer’s instructions. Biotin-labeled UL44 were resolved in SDS-PAGE, probed with Dylight 800 conjugated Streptavidin (Thermo Fisher Scientific) and analyzed by Odyssey CLx imaging system (Li-cor).

### Transfection-Replication Assays

HFFs plated on a 60-mm diameter dish were cotransfected with approximately 10 μg of total DNA containing plasmids listed in Table 2 encoding the HCMV core replication proteins, the auxiliary factors, and 1 μg pGL3-14 which contained the HCMV cloned oriLyt, using calcium phosphate co-precipitation method as described previously (Pari and Anders, 1993; Sarisky and Hayward, 1996). The same assay with HCMV UL44-K410A mutant was performed with the substitution of the plasmid pCMV-HA-UL44-K410A for the wild-type UL44 expression plasmid. A negative control assay was also performed the same as described above that all plasmids were cotransfected into HFFs, with the exception that the plasmid expressing UL54 was not added. In addition, SUMO-1 and UBC9 expression plasmids were also cotransfected with the above DNA mixtures to evaluate exogenous sumoylation effects. Total cellular DNA was harvested 5 days post-transfection using E.Z.N.A. Tissue DNA Kit (Omega Bio-Tek), and one-quarter of the total was cleaved with 30U *Dpn*I overnight and cleaned up by E.Z.N.A. Cycle Pure Kit (Omega Bio-Tek). The treated DNA was then used as template for the qPCR to determine the copies of oriLyt using primers 5′-GGGATTTGCCTGTGTATA-3′, 5′-CACGTCGTTTATCCTCAG-3′, and probe 5′-(FAM)CTC AGTCGCACAAGCAGCAC(BHQ-1)-3′. Human RnaseP p30 gene was also determined as a reference gene for normalization using primers 5′-AGATTTGGACCTGCGAGCG-3′, 5′-GAGCGGCTGTCTCCACAAGT-3′, and probe 5′-(FAM)TTCT GACCTGAAGGCTCTGCGCG(BHQ-1)-3′. The Premix Ex Taq Perfect Real Time Kit (Takara) was used for qPCR according to the manufacturer’s instructions and reactions were performed on an ABI 7500 real-time PCR system (Applied Biosystems). The standard curve for quantification of both oriLyt and RnaseP gene copies were constructed with 10-fold serial dilutions of pGL3-14 and a plasmid containing human RnaseP p30 gene. Each sample was done in triplicate. qPCR data were analyzed with 7500 real-time PCR software v2.0.5 (Applied Biosystems). Each set of assays was repeated 3 times and a representative one was shown.

### Dual-Luciferase Assay

Vero cells plated on a 60-mm diameter dish were cotransfected with plasmids encoding the HCMV core replication proteins (either with wild-type or K410A mutated UL44), the auxiliary factors, and pGL3-14 the same as described above in transfection-replication assay, in addition to 0.1 μg internal control reporter vector pRL-TK (Progema), using calcium phosphate co-precipitation method described previously (Sarisky and Hayward, 1996). A negative control assay was also performed the same as described above that all plasmids were cotransfected into Vero cells, with the exception that the plasmid expressing UL54 was not added. Firefly and renilla luciferase activity was measured with the use of a dual-luciferase reporter assay system (Promega) after total cell lysates were harvested 5 days post-transfection and processed as recommended by the manufacturer, and a Glomax 20/20 luminometer (Promega) was used to measure light emission. To confirm the conjugation of UL44 with SUMO-1, partial of cell lysates were directly probed with anti-HA antibody to visualize UL44. Analyses were repeated 3 times and the results were the average of triplicate experiments.

### Virus Generation

UL44 gene (wild-type or K410A mutant) was first subcloned from pCMV-HA-UL44 (wild-type or K410A mutant) to pEM7/Zeo vector (Invitrogen), UL44 together with the ZeoCassette which contains the Zeocin resistance marker was then amplified together by PCR. PCR products were used for generating Towne-BAC-UL44WT-Zeo and Towne-BAC-UL44-K410A-Zeo by Zeocin selection following a recombination-mediated method described before (Dunn et al., 2003), which introduces K410A mutation into the viral UL44 gene as well as ZeoR cassette right after UL44 loci in the strain Towne-BAC. To make a revertant virus, the Kanamycin cassette was amplified from pGBKT7 vector and replaced the ZeoCassette in pEM7/Zeo-UL44WT, resulting pKana-UL44WT. UL44 together with the Kana Cassette which contains the Kanamycin resistance marker was then amplified from pKana-UL44WT. PCR product was used for generating Towne-BAC-WT-Rev-Kana by Kanamycin selection. The insertion of K410A and Zeo or Kana cassette was confirmed by PCR and Southern blot. Original wild-type Towne-BAC, Towne-BAC-UL44WT-Zeo, Towne-BAC-UL44-K410A-Zeo, and Towne-BAC-WT-Rev-Kana were used to generate and propagate four HCMV viruses (WT, UL44WT-Zeo, UL44-K410A-Zeo, and WT-Rev-Kana) in HFF using the same method described before (Dunn et al., 2003).

### Sumoylation Analysis in Infected Cells

HFF cells were infected with wild-type HCMV (UL44WT-Zeo) or HCMV containing UL44 K410A mutant (UL44-K410A-Zeo) at an MOI of 1. At 48 h post-transfection, total cell lysates were either directly analyzed by SDS-PAGE, or immunoprecipitated with anti-UL44 antibody which was covalently immobilized on Protein A/G beads by Pierce Crosslink Magnetic IP/Co-IP Kit (Thermo Fisher Scientific), then probed for UL44 or sumoylated proteins using anti-UL44 (Santa Cruz Biotechnology) or anti-SUMO1 (Enzo Life Science) antibodies, respectively.

### Virus Yield Assay

Human primary foreskin fibroblasts (HFFs) (CC-2509) were obtained from Clonetics (San Diego) and propagated as described previously (Dunn et al., 2003). The replication and spread abilities of HCMV were determined by multi-step virus growth curves. The appropriate virus (WT, UL44WT-Zeo, UL44-K410A-Zeo, and WT-Rev-Kana) at a moi of 0.1 was added to a HFFs monolayer at 95% confluency. The inoculum was allowed to adsorb for 2 h, virus was removed and each well was washed with PBS. Supplemented DMEM was added to the cells, which were then incubated at 37°C. Cells were collected by scraping following infection at the indicated time points: 0, 2, 4, 6, 8, 10, 12, and 14 days. To release virus from infected cells, collected cells was frozen at −80°C and subsequently thawed at 37°C, this freeze-thaw cycle was repeated twice more. Infectious virus at each time point was titrated on HFFs. Virus was diluted 1:10 in DMEM supplemented with serum and further serial dilutions were performed for each growth curve. The appropriately diluted virus was added to HFFs and allowed to adsorb for 2 h, the virus was removed and DMEM supplemented with serum was added to each well. The infections were allowed to proceed for 14 days, and then plaques were counted which were identified by GFP expression. Titration of each time point was done in triplicate and titer values were means from triplicate experiments.

### Statistical Analysis

Data were analyzed using GraphPad Prism software and expressed as mean ± s.d. in figures. Differences between two groups were assessed by two-tailed unpaired Student’s *t*-test. Differences among three or more groups were assessed by One-way ANOVA with Tukey’s *post hoc* test. *P* < 0.05 was considered statistically significant.

## Results

### Identification of UBC9 as a UL44-Interacting Protein

To identify cellular proteins interacting with HCMV UL44, we used advanced GAL4-based yeast two-hybrid (YTH) system with yeast strain AH109 and three reporters (ADE2, HIS3, and lacZ). The reporters use distinct GAL4 upstream activating sequences and TATA boxes, which yield strong and specific responses to GAL4 protein. Full length UL44 was fused to the GAL4 DNA-binding domain (BD) as bait, and a human fetal brain cDNA library was fused to the GAL4 activation domain (AD) as preys. To virtually eliminate false positives, we used both ADE2 and HIS3 reporters which provided the strongest nutritional selection strength in the YTH system, together with lacZ for blue/white screening. Positive clones were selected on synthetic dropout medium lacking reporter nutrients (QDO) and tested for β-galactosidase activity by colony-lift filter assay. Screening of up to 2 × 10^7^ independent cDNA clones resulted in the isolation of 49 individual yeast colonies showing strong and specific responses to UL44. Sequence analysis then identified 36 out of 49 triple-positive clones as coding for either full-length or partial region of the human SUMO-conjugating enzyme UBC9 (Supplementary Figure 1, lane 1). Control experiments demonstrated that UL44 or UBC9 alone did not activate reporters (Supplementary Figure 1, lane 2–3), the co-expression of Lamin C and T antigen served as a negative control and the association of p53 and T antigen served as a positive control (Supplementary Figure 1, lane 4–5). Our results were consistent with a previous study using a LexA-based, two-reporter YTH system which also identified UBC9 as UL44 binding partner (Sinigalia et al., 2012).

**FIGURE 1:**
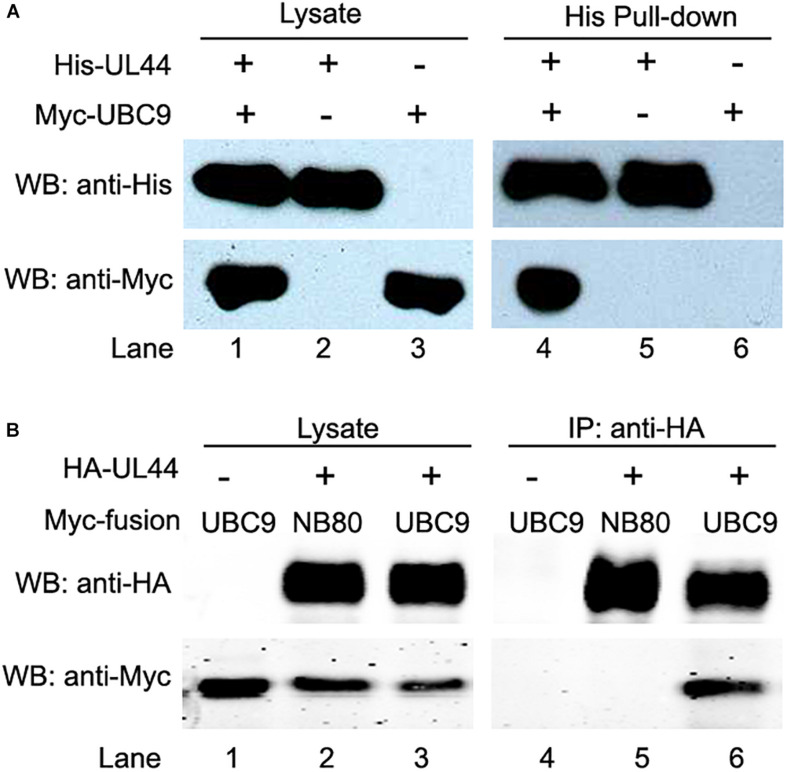
HCMV UL44 interacts with UBC9 *in vitro* and *in vivo*. **(A)** His-tagged UL44 pulled down Myc-tagged UBC9 *in vitro*. His-UL44 were bound to immobilized cobalt chelate resin then incubated with cell lysates expressing Myc-UBC9. The bound protein complexes were analyzed by western blot using anti-His and anti-Myc antibodies. Representative of three independent experiments. **(B)** Co-IP of transiently expressed UL44 and UBC9. 293T cells were cotransfected with HA-tagged UL44 and Myc-tagged UBC9 or NB80 as negative control. Cell lysates were incubated with immobilized anti-HA beads to isolate UL44 and its associated proteins. Results were analyzed by western blot using anti-HA and anti-Myc antibodies. Representative of three independent experiments.

In order to confirm that the observed interaction between UL44 and UBC9 in yeast is physiologically relevant in mammalian, first we sought to examine the subcellular location of UL44 and UBC9. When DsRed fused UBC9 or EGFP fused UL44 was transiently expressed in Hela cells alone, UBC9 had a nuclear and somewhat diffuse pattern, and UL44 formed a punctuate pattern in nuclei (Supplementary Figure 2). Co-expression of UL44 and UBC9 did not affect subcellular distributions of UBC9 nor UL44 (Supplementary Figure 2). Since both UL44 and UBC9 could locate in nuclei efficiently, fluorescent localization imaging might not be able to support UL44-UBC9 interaction convincingly. To address this problem, we next examined the interaction of UL44 with UBC9 by *in vitro* pull-down analysis. Immobilized cobalt chelate resin specifically isolated His-UL44 and its associated proteins when using bacteria-expressing His-tagged UL44 and mammalian cell-expressing Myc-tagged UBC9. Consistent with the YTH results, UBC9 specifically associated with His-UL44 (Figure 1A, lane 1 and 4), while no interaction with resin itself was observed (Figure 1A, lane 3 and 6).

The *in vivo* interaction between UL44 and UBC9 was then validated by co-IP test in 293T cells. Myc-UBC9 and HA-UL44 were transiently co-expressed and cell lysates were incubated with immobilized anti-Myc beads to isolate Myc-UBC9 and its associated proteins. HA-UL44 was co-IPed with Myc-UBC9 in a specific manner (Supplementary Figure 2). Finally, in order to demonstrate that UL44 does bind non-specifically to any highly expressed Myc-tagged protein in pull down and co-IP assays, Myc-tagged NB80, a nanobody only binds β-adrenoceptor and has similar molecular weight as UBC9, was used as negative control. Cell lysates co-expressing HA-UL44 and Myc-UBC9 or Myc-NB80 were incubated with immobilized anti-HA beads to isolate HA-UL44 and its associated proteins. As expected, we could detect the interaction of UBC9 (Figure 1B, lane 3 and 6), but not of NB80 (Figure 1B, lane 2 and 5), with HA-UL44. These observations demonstrate that UL44 and UBC9 formed a complex both *in vitro* and *in vivo*.

### Mapping the UL44 Region Required for the Interaction With UBC9

**FIGURE 2:**
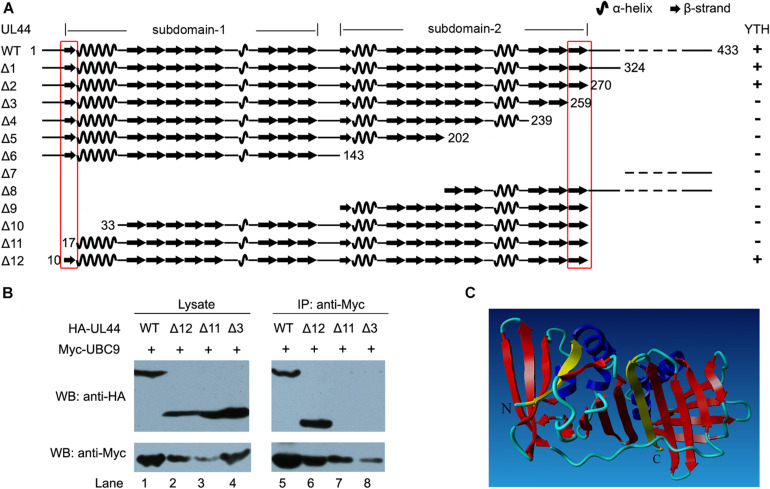
Mapping of the potential UBC9-interacting domains in UL44. **(A)** Schematic view and structural features of UL44 deletion mutants and their interaction with UBC9 as detected by yeast two-hybrid and β-galactosidase assays. +, positive interaction; –, no interaction. The first and last β-strands are indicated by red box. **(B)** Effect of different mutation on the interaction of UL44 with UBC9. 293T cells were cotransfected with Myc-tagged UBC9 and HA-tagged UL44 (full length or deletion mutants as indicated), then lysates were incubated with immobilized anti-Myc beads to isolate UBC9 and its associated proteins. **(C)** Stereoscopic ribbon representation of UL44 (aa 1–290) by YASARA view. UL44 aa 11–16 and 260–269, corresponding to the first and last β-strands, were colored yellow, was likely critical for the interaction of UL44 with UBC9. α-helices and other β-strands were colored blue and red, respectively.

To further explore the interaction between UL44 and UBC9, we sought to map the region of UL44 that was required for its interaction with UBC9. Full length UL44 contains 433 aa and the crystal structure of its processivity core (aa 1–290) was solved. This structure consists of two topologically similar subdomains connected by a loop (Appleton et al., 2004). A series of N- and C-terminal deletion derivatives of UL44 were made based on the secondary structure of UL44 (Figure 2A). Mutants were fused to GAL4-BD and then cotransformed with UBC9 fused with GAL4-AD into AH109 yeast strain for the YTH assays. We found that the truncated UL44Δ2 (aa 1–270), lacking the whole disordered C-terminal region of UL44, still exhibited interaction with UBC9. However, a further C-terminal truncated UL44Δ3 (aa 1–259), which removed the last β-strand of UL44 core (aa 260–270), exhibited no interaction with UBC9 anymore. On the other hand, the truncated UL44Δ12 (aa 10–270), lacking both the whole disordered C-terminal region and the small disordered N-terminal region of UL44, still exhibited interaction with UBC9. In contrast, a further N-terminal truncated UL44Δ11 (aa 17–270), which removed the first β-strand of UL44 core (aa 10–17), exhibited no interaction with UBC9 anymore.

Three UL44 deletion mutants (Δ3, Δ11, and Δ12) were further examined for their ability to bind UBC9 in mammalian cells by co-IP analysis. When UBC9 was immunoprecipitated, only full length UL44 (Figure 2B, lane 1,5) and UL44Δ12 (Figure 2B, lane 2, 6) were still associated with UBC9, while UL44Δ3 and Δ11 failed to do so. These data were consistent with YTH results and indicated that UL44Δ12 (aa 10–270), matched to UL44 processivity core with a well-ordered secondary structure, was the minimal UL44 derivative still being capable of interacting with UBC9. Although we could not further determine whether Δ3 and Δ11 affect UL44-UBC9 interaction through altering the overall folding of UL44 or losing direct contact regions with UBC9, our data suggested that the two small regions of UL44 (aa 11–16 and 260–269), which corresponded to the first and last β-strands of UL44 core, might be crucial for the interaction of UL44 with UBC9 (Figure 2C).

### UL44 Is a Target for Sumoylation

UBC9 mainly functions as the SUMO-conjugating enzyme, thus the observation that UL44 interacts with UBC9 prompted us to investigate the possibility that UL44 can be sumoylated. This hypothesis was tested both *in vitro* and *in vivo*.

**FIGURE 3:**
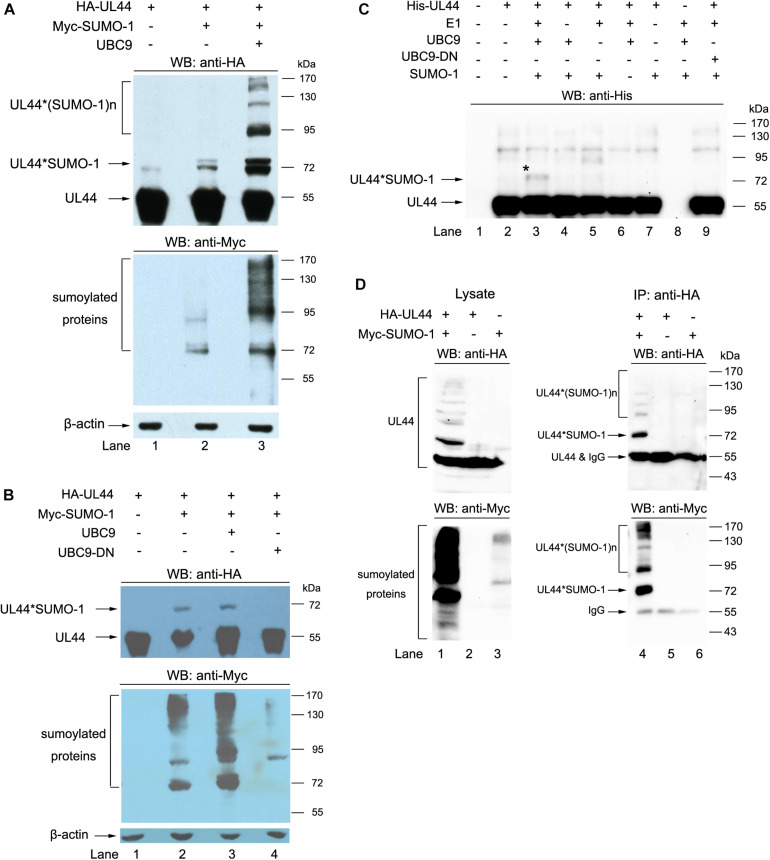
UL44 is a target for sumoylation. **(A)** UL44 covalently conjugated with SUMO-1. 293T cells were transiently cotransfected with HA-UL44, Myc-SUMO-1 and UBC9 as indicated. Forty eight hours later, cells were resuspended and boiled in SDS sample buffer, the supernatants were analyzed by western blot using anti-HA and anti-Myc antibodies. β-actin served as loading control. **(B)** UBC9 dominant-negative mutant abolished the sumoylation of UL44. 293T cells were cotransfected with HA-UL44, Myc-SUMO-1, UBC9, or UBC9-DN as indicated, and then processed the same as in **(A)**. **(C)** UL44 covalently conjugated with SUMO-1 *in vitro*. Purified His-tagged UL44, SUMO-activating E1 enzyme, UBC9, UBC9 dominant-negative mutant, and SUMO-1 were mixed in reaction buffer and incubated at 37°C for 2 h. The mixtures were separated by SDS-PAGE and UL44 was detected with anti-His antibody. **(D)** UL44 co-IPed with SUMO-1. Lysates from 293T cells expressing HA-UL44 and Myc-SUMO-1 were immunoprecipitated by anti-HA, the results were analyzed by western blot using anti-HA and anti-Myc antibodies. For all panels, representative of three independent experiments.

First, 293T cells cotransfected with HA-tagged UL44 and Myc-tagged SUMO-1 in the presence or absence of UBC9 were directly boiled and resolved on SDS-PAGE, and then probed for UL44 and sumoylated proteins by anti-HA and anti-Myc antibodies, respectively. SUMO-1 and UL44 migrate in SDS-PAGE at 17 and 52 kDa, respectively, thus a band about 70 kDa will form if UL44 is covalently conjugated by a SUMO-1 molecule. Results showed two UL44 bands migrating closely together around 72 kDa, which represent sumoylation of UL44 with the help of endogenous UBC9 (Figure 3A, lane 2) and additional exogenous UBC9 (Figure 3A, lane 3). The double bands might be caused by mono-sumoylation of different UL44 molecules at distinct lysine sites, which result in slightly different electrophoretic mobilities. A slight band occasionally appeared near 72 kDa in cells transfected with UL44 only, representing UL44 conjugated by an endogenous SUMO protein (Figure 3A, lane 1). Large sample load and long exposure time would yield additional UL44 species with different molecular weight roughly at 90 kDa, 110 kDa and higher [indicated as UL44^∗^(SUMO-1)n in Figure 3A], corresponding to one UL44 conjugated by two, three, and more SUMO-1 proteins, respectively, which suggested that UL44 could be multi-sumoylated on multiple lysines at the same time (Figure 3A, lane 3). This is consistent with previous report that UL44 could be extensively sumoylated at multiple sites (Sinigalia et al., 2012). Our results also showed that addition of exogenous UBC9 could significantly enhance mono- and multi-sumoylation of UL44 (Figure 3A, compare lane 2 and 3).

The SUMO-conjugating active site of UBC9 is centered on its ^93^cysteine, and mutation of ^93^cysteine to ^93^serine (C93S) would abolish the enzyme activity of UBC9 (Hay, 2005; Bossis and Melchior, 2006). To further confirm that sumoylation is responsible for the observed migrating change of UL44 in Figure 3A, we compared the difference of 293T cells transfected with HA-UL44 and Myc-SUMO-1, in the presence or absence of UBC9 or UBC9-C93S mutant. Over-expression of UBC9-C93S acted as a dominant-negative mutant (UBC9-DN) and severely impaired the function of endogenous UBC9, resulted in a significant decrease of sumoylated UL44 as well as endogenous cellular proteins (Figure 3B, compare lane 3 and 4).

The direct conjugation of UL44 with SUMO-1 was also confirmed by *in vitro* sumoylation assay. As expected, purified UL44 could be readily modified by SUMO-1 and a single mono-sumoylated product was observed *in vitro* (Figure 3C, lane 3). The appearance of slower migrating form of UL44 by sumoylation was abolished if either SUMO-1, E1, UBC9 was omitted from the reaction (Figure 3C, lane 4–6), or UBC9 dominant-negative mutant was used (Figure 3C, lane 9). Unlike our *in vivo* tests and a previous study showing multi-sumoylated UL44, we could only detect mono-sumoylated UL44 in the *in vitro* assay. There were many facts probably influenced the test, such as incubation time, temperature, activity of enzymes, different prokaryotic forms of UL44 (e.g., GST or His tag), or ratio between UL44 and other molecules in the reaction.

Finally, we used co-IP test to confirm the conjugation of SUMO-1 with UL44. 293T cells were transiently transfected with HA-UL44 and Myc-SUMO-1 alone or together. UL44 in cell lysates were immunoprecipitated and its associated proteins were probed with anti-HA and anti-Myc antibodies. Same as in Figures 3A,B, we observed multiple SUMO bands in UL44 immunoprecipitation (Figure 3D, lane 4). Taken together, the results above suggested that UL44 was a target for sumoylation and was covalently modified by SUMO-1.

### ^410^lysine Is the Major Sumoylation Site of UL44

UL44 contains 31 lysines, and our results have shown that UL44 could be mono- and multi-sumoylated. Using online sumoylation sites prediction software (Ren et al., 2009) we found that UL44 contained only one high-probability ψKxE sumoylation site at its ^410^lysine. To determine whether UL44 was sumoylated at this consensus lysine, we mutated ^410^lysine to ^410^alanine (K410A). Because alanine is natural and small, and aa 410 is at the very end of UL44 where is disordered in native UL44, K410A mutation would have minimal impacts on protein structure. We did not use conservative arginine substitution for ^410^lysine in order to avoid introducing other post-translational modifications such as methylation potentially.

**FIGURE 4:**
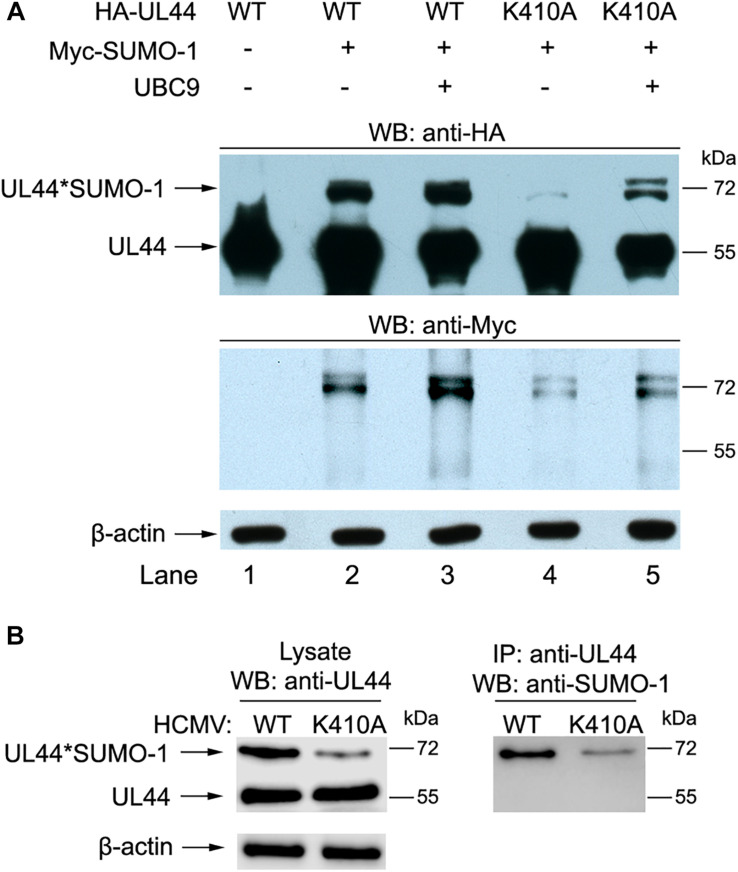
^410^lysine was the major sumoylation site of UL44 in both transfected and infected cells. **(A)** 293T cells were transiently cotransfected with HA-UL44 (wild-type or K410A mutant), Myc-SUMO-1 and UBC9 as indicated. Forty eight hours later, cells were resuspended and boiled in SDS sample buffer, the supernatants were analyzed by western blot using anti-HA and anti-Myc antibodies. **(B)** HFF cells were infected with wild-type HCMV (WT) or HCMV containing UL44 K410A mutant (K410A) at an MOI of 1. At 48 h post-transfection, total cell lysates were either directly analyzed by SDS-PAGE, or immunoprecipitated with covalently conjugated anti-UL44 beads, then probed for UL44 or sumoylated proteins using anti-UL44 or anti-SUMO1 antibodies, respectively. For all panels, representative of three independent experiments.

We examined sumoylation of UL44-K410A mutant with SUMO-1. Western blot results showed UL44-K410A could still be sumoylated, but the removal of ^410^lysine site resulted in substantially reduced sumoylation (Figure 4A, compare lane 2 and 4), and the addition of UBC9 would only partially recover UL44 sumoylation capability (Figure 4A, compare lane 3 and 5). This suggested that ^410^lysine within the ψKxE consensus motif was the major sumoylation site of UL44, and other lysine residues can also be sumoylated to a much lesser extent.

We then tested if this was also the case in virus. HFF cells were infected with wild-type HCMV (WT) or HCMV containing UL44 K410A mutant (K410A), total cell lysates were either directly analyzed for UL44 or sumoylated proteins using anti-UL44 or anti-SUMO1 antibodies, or immunoprecipitated with covalently conjugated anti-UL44 beads then probed with anti-SUMO1 antibodies. Consistently, UL44-K410A virus could still be sumoylated, but the removal of ^410^lysine site resulted in substantially reduced sumoylation (Figure 4B). These data suggested that ^410^lysine was the major sumoylation site of UL44 in both transfected and infected cells.

### Mutation of ^410^lysine Had No Effects on Subcellular Localization or Protein Stability of UL44

**FIGURE 5:**
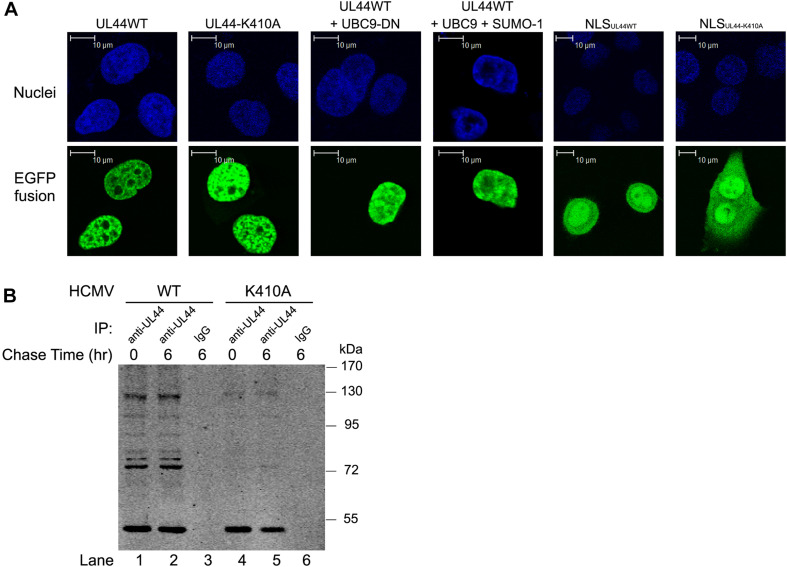
^410^lysine had no effects on subcellular localization or protein stability of UL44. **(A)** Hela cells were transfected with constructs encoding fusion proteins EGFP-UL44WT, EGFP-UL44-K410A, EGFP-NLS_*UL*44*WT*_, EGFP-NLS_*UL*44–*K*410*A*_, and UBC9 or SUMO-1 alone or together as indicated in figure, stained with Hoechst 33258 to visualize cell nuclei. Confocal microscopy showed mutation of ^410^lysine had no effect on the nuclear localization of UL44. **(B)** HFFs were infected with wild-type HCMV (WT) or HCMV carrying UL44-K410A mutant (K410A) for 48 h. Cells were then pulsed in methionine-free DMEM with methionine analog AHA. After pulse labeling, cells were either immediately harvested (0 h), or chased in regular DMEM for 6 h (6 h). Cell lysates containing AHA-incorporated proteins were then immunoprecipitated with anti-UL44 antibody and further cross-linked with biotin. Biotin-labeled UL44 were resolved and visualized in SDS-PAGE. Pulse-chase test showed mutation of ^410^lysine had no effects on protein stability of UL44.

Sumoylation has diverse functions in regulating many cellular pathways, such as cell cycle, transcription, gene expression, chromatin structure, RNA metabolism. Two of most common biological effects exerted by sumoylation are stabilizing proteins and protecting them from degradation, as well as affecting cellular localization. Since DNA processivity function of UL44 happened in the nuclear, we first tested whether sumoylation played any role in the nuclear localization of UL44. Fluorescent localization studies on the wild-type and K410A mutated UL44 fused with EGFP in Hela cells showed that both proteins formed the same punctuate pattern in nuclei (Figure 5A, column 1 and 2). Neither co-expression of UBC9-DN which abolished sumoylation nor co-expression of SUMO-1 and UBC9 which enhanced sumoylation could alter the location of UL44 (Figure 5A, column 3 and 4). To further confirm this, we also investigated the nuclear targeting function of the extreme C terminus of UL44, which was the last 29 amino acids of UL44 containing the ^410^lysine sumoylation motif and NLS motif. The expression of EGFP-NLS_*UL*44w*t*_ and EGFP-NLS_*UL*44–*K*410*A*_ showed that both the wild-type short signal peptide and its K410A mutant were sufficient to direct EGFP protein to nuclei (Figure 5A, column 5 and 6). These observations suggested that mutation of ^410^lysine had no effect on the nuclear localization of UL44.

We then tested whether sumoylation played any role in the stability of UL44. HFFs were infected with wild-type HCMV (WT) or HCMV carrying UL44-K410A mutant (K410A) for 48 h. Cells were then pulsed in methionine-free DMEM with methionine analog AHA, which specifically labeled de novo synthesized proteins. After pulse labeling, cells were either immediately harvested (0 h), or chased in regular DMEM for 6 h (6 h) when de novo synthesized proteins underwent physiological degradation. Cell lysates containing AHA-incorporated proteins were then immunoprecipitated with anti-UL44 antibody and further cross-linked with biotin. Biotin-labeled UL44, which exclusively represented de novo synthesized UL44 during pulse period, was resolved and visualized in SDS-PAGE. Pulse-chase test showed that UL44 was stable and had minimal degradation during 6-h chase period (Figure 5B, compare lane 1 and 2). More importantly, mutation of ^410^lysine did not alter the stability of UL44 (Figure 5B, compare lane 4 and 5). Additionally, the multiple migrating bands of UL44 in the assay were consistent with the predicted molecule weight of UL44 with mono- and multi-sumoylation, and K410A mutation severely impaired such modification (Figure 5B, compare lane 1 and 4), although we could not exclude other types of post-modification on UL44.

### Mutation of UL44 ^410^lysine Enhanced OriLyt DNA Synthesis in Transfection-Replication Assays

Although mutation of ^410^lysine had no effects on subcellular localization or protein stability of UL44, it indeed hampered UL44 sumoylation. To gain some insights on the role of UL44 sumoylation in the context of HCMV DNA replication, we employed several different approaches. It was shown previously that HCMV core replication proteins along with certain auxiliary factors could support efficient DNA amplification of cloned HCMV oriLyt (lytic-phase DNA replicator) in a transient transfection-replication system, either in HCMV permissive cells like HFFs or non-permissive cells like Vero cells (Pari and Anders, 1993; Sarisky and Hayward, 1996). To investigate whether the mutation of UL44 ^410^lysine has any effect on viral DNA replication, similar assays were adopted in this study. The oriLyt-containing plasmid pGL3-14, which contained the *Pvu*II/*Kpn*I segment of HCMV oriLyt and a cDNA encoding firefly luciferase under SV40 promoter, served as an assayable reporter of *in vitro* oriLyt-mediated DNA synthesis. The plasmids described in Table 2 were cotransfected into either HFFs or Vero cells along with pGL3-14, and then total DNA from transfected cells were harvested and measured by two different methods in order to evaluate oriLyt copies quantitatively. A negative control was also performed that all other plasmids were cotransfected with the exception of viral DNA polymerase UL54. In some cases, SUMO-1 and UBC9 were also introduced into the transfection mixture to evaluate exogenous sumoylation effects.

**FIGURE 6:**
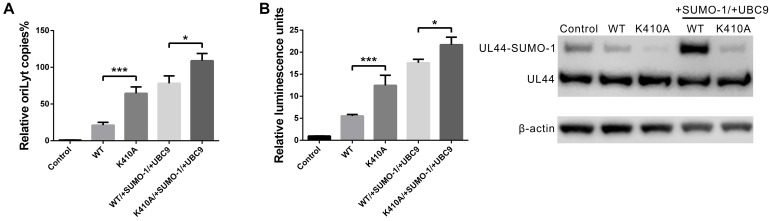
Mutation of UL44 ^410^lysine enhanced oriLyt-dependent DNA synthesis in transfection-replication assays. **(A)** HFF cells were transiently cotransfected with pCMV-HA-UL44 (wild-type or K410A mutant) and all other plasmids as indicated in Table 2, or without UL54 expression plasmid as control. SUMO-1 and UBC9 expression plasmids were also cotransfected in certain groups as indicated. Total cellular DNA was harvested 5 days post-transfection and was cleaved with *Dpn*I, and then was used as qPCR template to determine the copies of oriLyt-containing plasmid pGL3-14. Each sample was done in triplicate. Each set of assays was repeated 3 times and a representative one was shown. **(B)** Vero cells were transiently cotransfected with HA-UL44 (wild-type or K410A mutant) and all other plasmids as indicated in Table 2, or without UL54 expression plasmid as control. SUMO-1 and UBC9 expression plasmids were also cotransfected in certain groups as indicated. Luciferase activity was measured 5 days after transfection. Firefly luciferase activity was normalized to Renilla luciferase activity for each sample. To confirm the conjugation of UL44 with SUMO-1, partial of cell lysates were directly probed with anti-HA antibody to visualize UL44. Analyses were repeated 3 times and the results were the average of triplicate experiments. Error bars = SD. * denotes *p* < 0.05, and *** denotes *p* < 0.001 by two-tailed unpaired Student’s *t*-test between indicated two groups.

First, quantitative real-time PCR (qPCR) was used to determine replicated copies of pGL3-14 in transfected HFFs. Primers and probes were designed to produce PCR products either spanning a 158 bp oriLyt fragment containing an endogenous *Dpn*I cleavage site, or a 65 bp sequence located in RnaseP p30 gene for normalization purpose. Plasmids produced from bacteria were Dam-methylated and *Dpn*I-sensitive, therefore after *Dpn*I treatment all transfected progenitor plasmids were cleaved, while newly synthesized progeny pGL3-14 and cellular DNA were non-methylated thus *Dpn*I-resistant and could be detected by qPCR. Our results showed that the replicated copies of oriLyt in UL44-K410A group were roughly threefolds more than that of UL44-WT group (Figure 6A). Introduction of SUMO-1 and UBC9 promoted more replicated oriLyt, and K410A mutation further boosted the replication.

Second, dual-luciferase assay was used as an alternative way to monitor replication level of pGL3-14 in transfected Vero cells. The use of Vero cells offered the opportunity to cotransfect the set of replication plasmids in a more efficient manner than in HFFs. pGL3-14 constructed in this study was confirmed to constitutively express firefly luciferase in mammalian cells, when other experimental conditions were constant, the levels of firefly luciferase activity which correlated with pGL3-14 gene expression, reflected the total amounts of pGL3-14 in cells. pGL3-14 was cotransfected into Vero cells together with the Renilla luciferase-expressing plasmid pRL-TK as a normalizing control, and other replication assay plasmids in the presence of wild-type or K410A UL44. Our results showed that the luciferase activity in Vero cells transfected with UL44-K410A was roughly twofolds higher than that of the parallel transfected cells using wild-type UL44 (Figure 6B), indicating more copies of pGL3-14 in K410A group than WT group. Consistent with qPCR results, introduction of SUMO-1 and UBC9 promoted the luciferase activity in UL44-WT group, and K410A mutation further boosted the activity. In the same assay, to confirm the conjugation of UL44 with SUMO-1, partial of cell lysates were directly probed with anti-HA antibody to visualize UL44 and its modified forms (Figure 6B). The results collected from qPCR and luciferase assays indicated that pGL3-14 was efficiently replicated in HFFs and Vero cells, and the mutation of ^410^lysine enhanced oriLyt DNA synthesis in transfection-replication assays.

### Mutation of UL44 ^410^lysine Enhanced Progeny Production of HCMV Infection in HFFs

**FIGURE 7:**
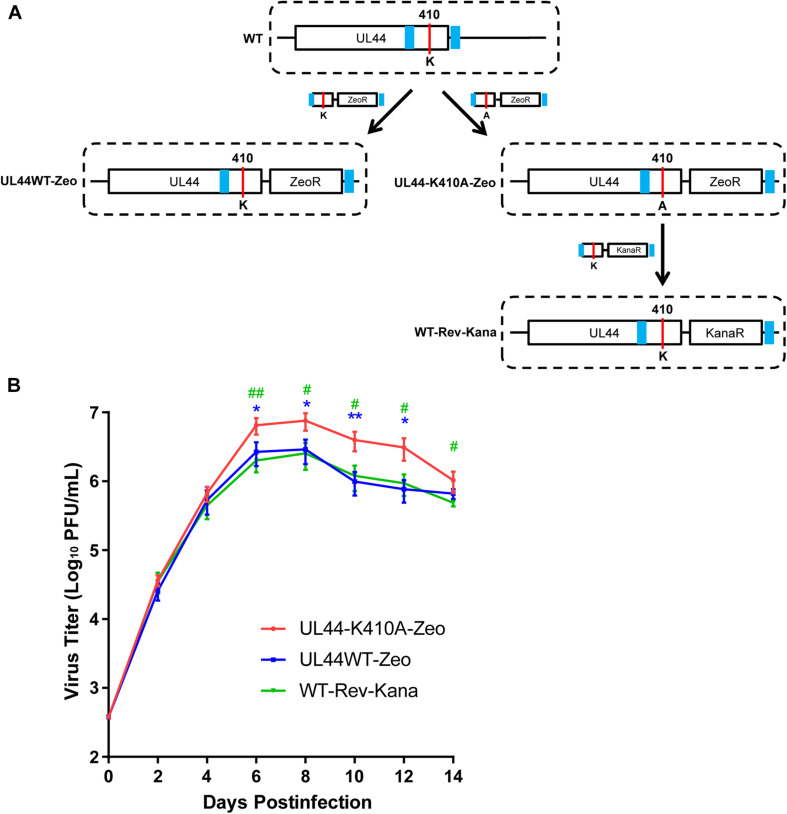
Mutation of ^410^lysine enhanced the viral replication of HCMV in HFFs. **(A)** Schematic for BAC construction and generation of wild-type, mutant and revertant HCMV virus. **(B)** The replication and spread abilities of HCMV were determined by multi-step virus growth curves. HFF cells were infected by appropriate virus (UL44WT-Zeo, UL44-K410A-Zeo, and WT-Rev-Kana) at a moi of 0.1 and collected at the indicated time points. Released virus at each time point was titrated on HFFs by serial dilutions and plaques counting to generate growth curves. Titration of each time point was done in triplicate and titer values were means from triplicate experiments. Error bars = SD. Differences among three viruses at each time point were assessed by One-way ANOVA with Tukey’s *post hoc* test. * denotes *p* < 0.05, and ** denotes *p* < 0.01 by One-way ANOVA with Tukey’s multiple comparison test with UL44WT-Zeo. # denotes *p* < 0.05, and ## denotes *p* < 0.01 by One-way ANOVA with Tukey’s multiple comparison test with WT-Rev-Kana.

The finding that mutation of ^410^lysine enhanced oriLyt DNA synthesis in transfection-replication assays promoted the idea that sumoylation could attenuate HCMV replication through targeting UL44. Since such plasmid-based assays might not directly infer what happen during viral DNA synthesis in the infected cell, we further tested whether the alteration on UL44 sumoylation would affect the viral progeny replication efficiency *in vivo*. As other HCMV proteins (e.g., IE1 and IE2) can be sumoylated and influence viral replication, instead of over-expressing SUMO-1, we used mutant viruses which allow us directly dissect SUMO effects on UL44 only. To make a HCMV virus containing UL44-K410A single-site mutation, ZeoR cassette was introduced right after the UL44 loci on the Towne-BAC for the recombination selection purpose, and KanaR was used for making the revertant virus (Figure 7A). Four viruses were made: WT generated from original wild-type Towne-BAC, UL44WT-Zeo, and UL44-K410A-Zeo generated by Towne-BAC-UL44WT-Zeo and Towne-BAC-UL44-K410A-Zeo which contains ZeoR cassette right after UL44, and WT-Rev-Kana as the revertant version of UL44-K410A-Zeo.

The titers of viral particles produced from HFF cells infected with different viruses were determined and compared. Interestingly, we first observed higher viral titers when comparing UL44WT-Zeo or WT-Rev-Kana with WT (Supplementary Figure 3). This suggests the insertion of Zeocin or Kanamycin resistance gene at downstream of UL44 somehow impacts the virus replication in some way. To exclude such impacts, we then only compared UL44WT-Zeo, UL44-K410A-Zeo, and WT-Rev-Kana in the growth curve. Starting from day-6 post-infection, UL44-K410A-Zeo virus containing K410A single-site mutation produced significantly higher viral titers (2.5–4-fold changes) than UL44WT-Zeo and WT-Rev-Kana (Figure 7B), indicating the removal of ^410^lysine sumoylation site could significantly enhance the viral production. Together, these results suggested that mutation of UL44 ^410^lysine enhanced progeny production of HCMV infection in HFFs. Consequently, sumoylation of the carboxy-terminal of human cytomegalovirus DNA polymerase processivity factor UL44 attenuates viral DNA replication.

## Discussion

UL44 is an essential gene of HCMV and plays its key role in viral DNA replication. To better understand the biological function of UL44, we performed a yeast two-hybrid screen and the most frequently isolated UL44-interacting protein was the SUMO-conjugating enzyme UBC9. The interaction of UL44 with UBC9 was confirmed both *in vitro* and *in vivo*. Consistent with our findings, other group also reported the interaction between UL44 and UBC9 (Sinigalia et al., 2012). Our deletion analysis then located two small β-strands of UL44 core (aa 11–16 and 260–269) critical for the interaction with UBC9. However the previous study suggested that UL44 binding domains to UBC9 were likely within aa 1–200 and 313–433 (Sinigalia et al., 2012). These inconsistent observations are likely due to different truncation strategy. The previous study did large fragment truncation while our study truncated UL44 based on its secondary structure in a small deletion manner, theoretically with fewer impacts on the overall folding of UL44. The differences could also arise from YTH screening strength: the previous study used a two-reporter system and we used a three-reporter system. A crystal structure of UL44-UBC9 complex in the future will ultimately help us to reveal the precise docking regions between UL44 and UBC9.

Before our study, there are two contrary reports regarding whether UL44 can be sumoylated (Sinigalia et al., 2012; Kim et al., 2014). Our studies confirmed that UL44 was a target for sumoylation and could be effectively conjugated with SUMO-1. We further determined ^410^lysine, located at the extreme carboxy terminus of UL44 and within a ψKxE consensus sumoylation motif, as the major sumoylation site of the protein. Mutation of ^410^lysine at UL44 carboxy-terminal strongly but not completely reduced the sumoylation capability of UL44, suggesting that the lysine within this ψKxE consensus motif is the major sumoylation site of UL44, and there are non-consensus lysines serving as sumoylation sites also. More importantly, although the mutation of ^410^lysine had no effects on subcellular localization or protein stability of UL44, we found that the removal of ^410^lysine sumoylation site enhanced both viral DNA synthesis in transfection-replication assays and viral progeny production in infected cells for HCMV, indicating a negative modulation of sumoylation on HCMV DNA replication and virus production.

Consistent with our findings, the previous study also reported UL44 could be sumoylated at multiple sites. In contrast to our findings, the previous study failed to identify a major sumoylation site (Sinigalia et al., 2012). We notice that the previous study used *in vitro* assay and an *E. coli* modification system as the main tools to determine sumoylation sites in UL44, which not necessarily reflect the actual UL44 modification *in vivo*. This notion is exemplified by the fact that a later study used exactly the same *E. coli* modification system but failed to detect any UL44 sumoylation (Kim et al., 2014).

The previous study showed that over-expression of SUMO-1 enhances both virus production and DNA replication in HCMV-infected cells (Sinigalia et al., 2012), which seems to be contrary to our results. However, it should be noted that previous work was done by wild-type HCMV infection and global SUMO-1 over-expression in cells, while our study compared wild-type HCMV with site-specific UL44 mutants and directly dissected the SUMO effects on UL44 only. More importantly, since the viral IE1/IE2 activity are well-known to be up-regulated upon sumoylation, the observation that SUMO-1 over-expression causes a positive effect on HCMV replication could be due to the enhancement of IE1/IE2 activity (Nevels et al., 2004; Reuter et al., 2017), or due to affecting cell physiology and host immune defense. Indeed, in the same report it has shown that SUMO-1 might slightly alters the intranuclear distribution of UL44 (Sinigalia et al., 2012), this result is consistent with our finding of negative modulation of sumoylation on UL44 functions.

Sumoylation has been identified as an important cellular regulation process in protein function, thus the interplay between viruses and sumoylation might be common. Previous reports showed viral proteins that either can be sumoylated or influence the sumoylation pathway are generally encoded by herpesviruses and belong to immediate-early proteins, and sumoylation of these proteins is believed to be a way for herpesviruses to regulate their transcription activation (Muller and Dejean, 1999; Hofmann et al., 2000; Ahn et al., 2001; Gravel et al., 2002; Chang et al., 2004; Nevels et al., 2004; Adamson, 2005; Izumiya et al., 2005; Boggio and Chiocca, 2006; Gan et al., 2016; Lin et al., 2017; Reuter et al., 2017; Jan Fada et al., 2020). UL44 mainly involves in viral DNA replication and it is not a herpes immediate-early protein, implying sumoylation can play distinct roles throughout the process of herpesviral infection.

**FIGURE 8:**
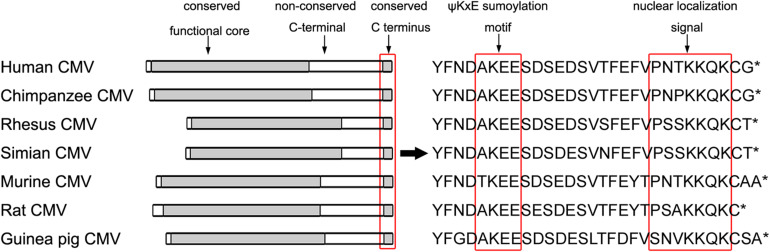
UL44 homologs share a highly conserved C terminus. Nuclear localization signals are indicated by red box. Putative ψKxE consensus sumoylation motifs corresponding to HCMV ^410^lysine are indicated in boldface type and by red box. The single-letter amino acid code is used and * means the end of a protein.

It is yet not clear whether the repression of HCMV viral replication via UL44 sumoylation is an intrinsic cellular immunity against HCMV infection, or a self-restricted strategy of virus for optimizing its replication in host cells and for persistent infections (Dunn et al., 2003). Previous studies showed that the catalytic activities reside in the amino-terminal region of UL44, meanwhile little is known about the role of the carboxy-terminal segment of UL44, although it was found to be indispensable for virus replication and for the formation of DNA replication compartments in infected cells (Kim and Ahn, 2010; Silva et al., 2010). Sequence alignments revealed that UL44 and its CMV homologs share a conserved amino-terminal functional core, a non-conserved central region covering most of carboxy-terminal, and intriguingly, a highly conserved tail located at the extreme carboxy terminus containing the last 29 amino acids (Figure 8). Since CMV viruses are highly species-specific, the evolutionary conservation of the tail across UL44 and its CMV homologs is most relevant as an evidence for how herpesviruses utilize cellular pathways to achieve optimal persistent infections within the host through self-restricted replication.

Here we identified the highly conserved very last 29 amino acids containing the ^410^lysine as well as NLS, strongly support the idea that HCMV harbors the ability of sumoylation on UL44 for the self-restricted purpose. This also clearly demonstrates a crucial role for the carboxy-terminal of UL44 in HCMV viral DNA synthesis and viral replication. Suppression of viral replication by sumoylation represents a novel mechanism for regulating the growth of herpesviruses, and may contribute to HCMV optimal infection of different tissues and successful proliferation among the human population. It is also possible that this segment has some other roles in viral replication and/or persistent infections, perhaps in gene expression or interactions with host or viral proteins, but not yet experimentally demonstrated. Thus, our findings could inspire further studies on sumoylation of herpesviruses and, more in general, of other viruses.

## Data Availability Statement

The original contributions presented in the study are included in the article/Supplementary Material, further inquiries can be directed to the corresponding author/s.

## Author Contributions

JC and AS conceived and designed the study. JC, GL, HH, XL, WN, and DC performed the experiments. JC and AS analyzed the data, advised on the experiments, contributed reagents, materials, analysis tools, and wrote the manuscript. All authors read and approved the final manuscript.

## Conflict of Interest

The authors declare that the research was conducted in the absence of any commercial or financial relationships that could be construed as a potential conflict of interest.

## Supplementary Material

Supplementary Figure 1 Interaction of UL44 with UBC9 as analyzed by yeast two-hybrid and β-galactosidase assays. The association of p53 and T antigen served as a positive control and the co-expression of Lamin C and T antigen served as a negative control.

Supplementary Figure 2 Both UL44 and UBC9 located in nuclei. Hela cells were transfected with constructs encoding EGFP-UL44 and DsRed-UBC9 alone or together, stained with Hoechst 33258 to visualize cell nuclei. UL44 alone located in nuclei and UBC9 alone mainly located in nuclei.

Supplementary Figure 3 Co-IP of transiently expressed UL44 and UBC9. 293T cells were cotransfected with HA-tagged UL44 and Myc-tagged UBC9 as indicated. Cell lysates were incubated with immobilized anti-Myc beads to isolate UBC9 and its associated proteins. Results were analyzed by western blot using anti-HA and anti-Myc antibodies. Representative of three independent experiments.

Supplementary Figure 4 Effects of antibiotics resistance cassette insertion on the viral replication of HCMV in HFFs. **(A)** Schematic for BAC construction and generation of wild-type, mutant and revertant HCMV virus. **(B)** The replication and spread abilities of HCMV were determined by multi-step virus growth curves. HFF cells were infected by appropriate virus (WT, UL44WT-Zeo, UL44-K410A-Zeo, and WT-Rev-Kana) at a moi of 0.1 and collected at the indicated time points. Released virus at each time point was titrated on HFFs by serial dilutions and plaques counting to generate growth curves. Titration of each time point was done in triplicate and titer values were means from triplicate experiments. Note that introduction of antibiotics resistance cassette downstream of UL44 loci could enhance the viral replication comparing to WT HCMV.
