# Supplementary figures and images for: Sumoylation of the Carboxy-Terminal of Human Cytomegalovirus DNA Polymerase Processivity Factor UL44 Attenuates Viral DNA Replication
Source: Front Microbiol. 2021 Apr 21;12:652719. doi: 10.3389/fmicb.2021.652719 (PMC8097051; doi:10.3389/fmicb.2021.652719)

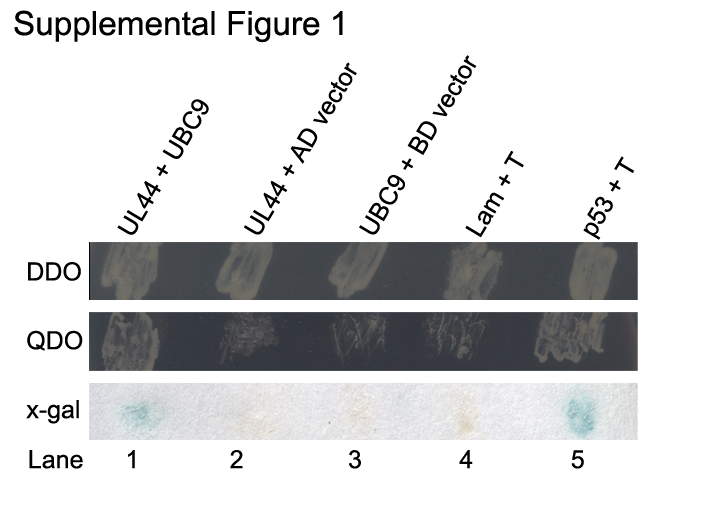

Supplement: Supplementary Figure 1 — Interaction of UL44 with UBC9 as analyzed by yeast two-hybrid and β-galactosidase assays. The association of p53 and T antigen served as a positive control and the co-expression of Lamin C and T antigen served as a negative control. [file Image_1.tif]

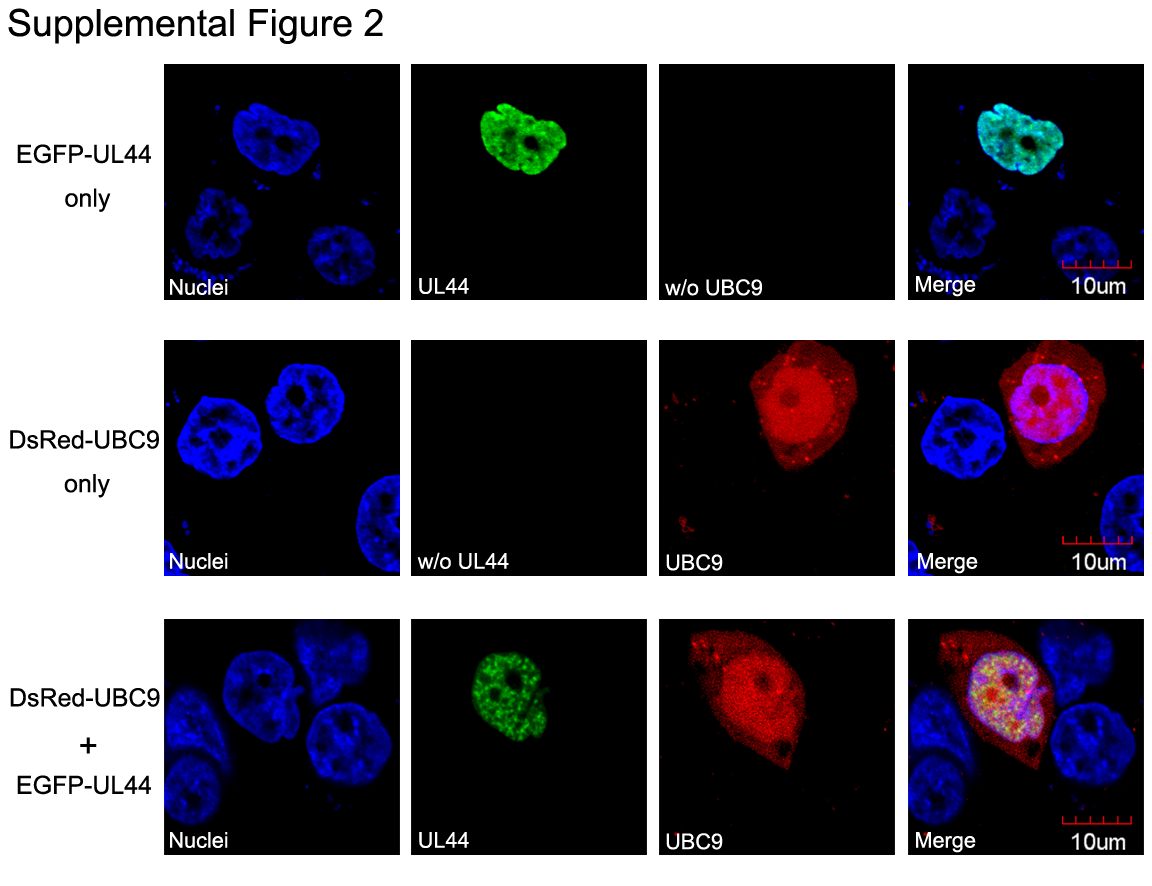

Supplement: Supplementary Figure 2 — Both UL44 and UBC9 located in nuclei. Hela cells were transfected with constructs encoding EGFP-UL44 and DsRed-UBC9 alone or together, stained with Hoechst 33258 to visualize cell nuclei. UL44 alone located in nuclei and UBC9 alone mainly located in nuclei. [file Image_2.tif]

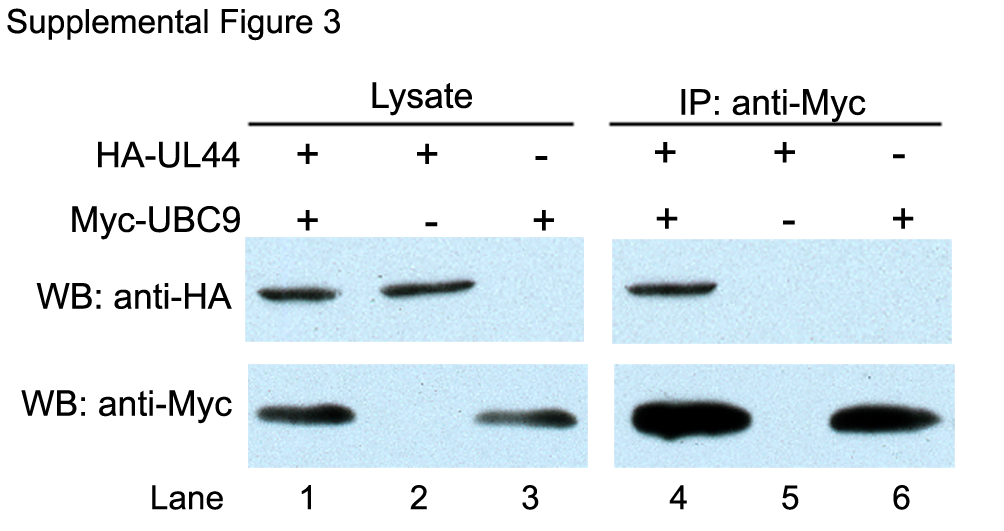

Supplement: Supplementary Figure 3 — Co-IP of transiently expressed UL44 and UBC9. 293T cells were cotransfected with HA-tagged UL44 and Myc-tagged UBC9 as indicated. Cell lysates were incubated with immobilized anti-Myc beads to isolate UBC9 and its associated proteins. Results were analyzed by western blot using anti-HA and anti-Myc antibodies. Representative of three independent experiments. [file Image_3.tif]

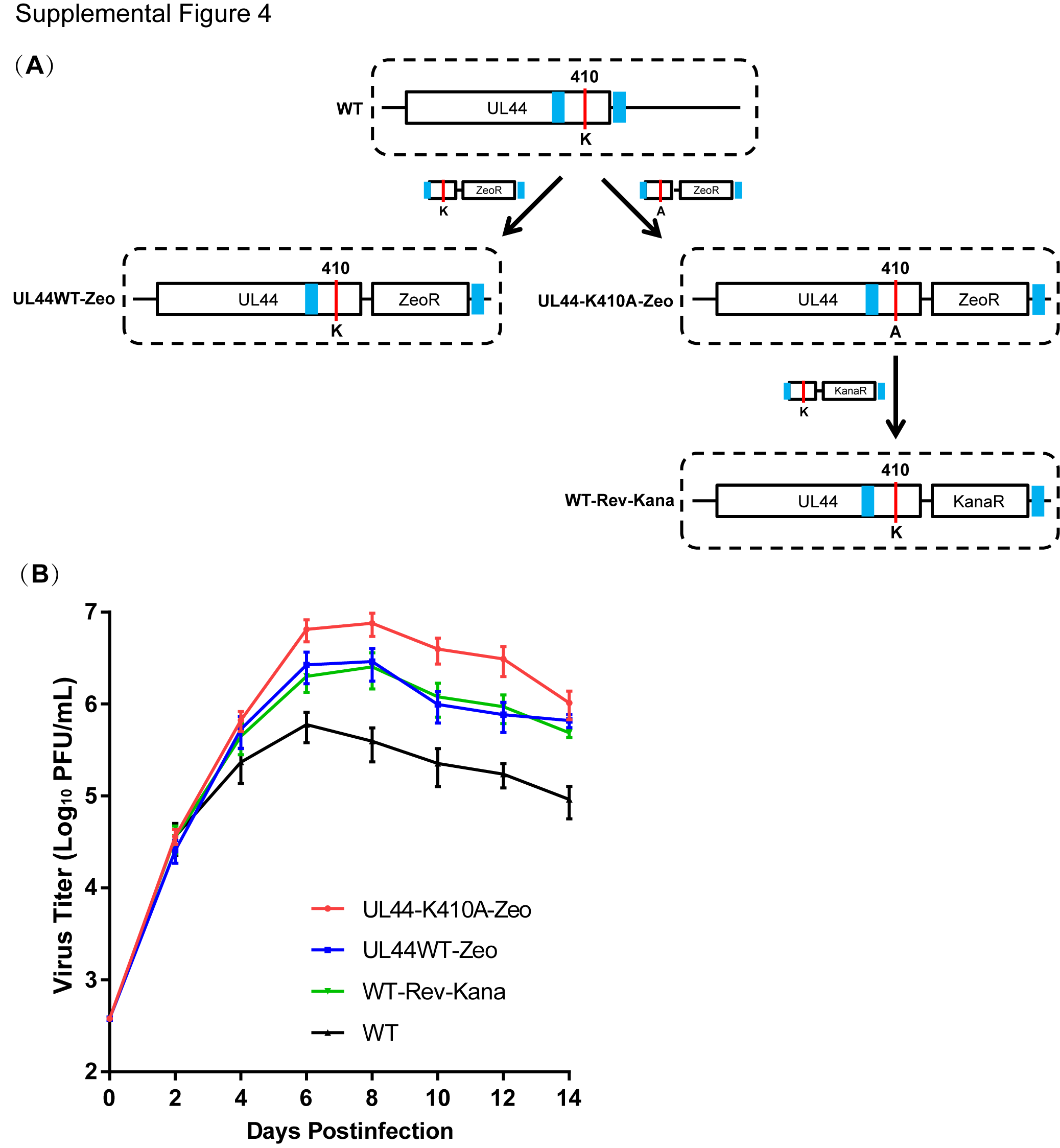

Supplement: Supplementary Figure 4 — Effects of antibiotics resistance cassette insertion on the viral replication of HCMV in HFFs. (A) Schematic for BAC construction and generation of wild-type, mutant and revertant HCMV virus. (B) The replication and spread abilities of HCMV were determined by multi-step virus growth curves. HFF cells were infected by appropriate virus (WT, UL44WT-Zeo, UL44-K410A-Zeo, and WT-Rev-Kana) at a moi of 0.1 and collected at the indicated time points. Released virus at each time point was titrated on HFFs by serial dilutions and plaques counting to generate growth curves. Titration of each time point was done in triplicate and titer values were means from triplicate experiments. Note that introduction of antibiotics resistance cassette downstream of UL44 loci could enhance the viral replication comparing to WT HCMV. [file Image_4.tif]
